# Outcomes of professional misconduct by nurses: a qualitative study

**DOI:** 10.1186/s12912-024-01859-3

**Published:** 2024-03-25

**Authors:** Shokoh Varaei, Nahid Dehghan Nayeri, Leila Sayadi, Mehraban Shahmari, Akram Ghobadi

**Affiliations:** 1 School of Nursing and Midwifery, Tehran University of Medical Sciences, Tehran, Iran; 2 Nursing and Midwifery Care Research Center, School of Nursing and Midwifery, Tehran University of Medical Sciences, Tehran, Iran; 3 School of nursing & midwifery, Nursing and Midwifery Care Research Center, Tehran University of Medical Sciences, Tehran, Iran; 4 Department of Medical-Surgical, School of Nursing and Midwifery, Ardabil University of Medical Sciences, Ardabil, Iran; 5 School of Nursing and Midwifery, Kermanshah University of Medical Sciences, Kermanshah, Iran

**Keywords:** Professional misconduct, Nurses, Qualitative research

## Abstract

**Background:**

Professional misconduct by nurses is a critical challenge in providing safe quality care, which can lead to devastating and extensive outcomes. Explaining the experiences of clinical nurses and nursing managers in this regard using an in-depth qualitative method can be beneficial. This study was conducted with the aim of explaining the experiences of nurses regarding the outcomes of professional misconduct.

**Methods:**

The present study used a qualitative descriptive with a conventional content analysis approach. A total of 22 clinical nurses and nursing managers were selected through purposive sampling until data saturation was reached. Data were collected using semi-structured in-depth interviews and analyzed using Graneheim and Lundman’s approach.

**Results:**

Analyzed data were categorized into four main themes and 11 subthemes: (1) Physical outcomes: critical threat and weakening patients’ safety; (2) Psychological outcomes: psycho-emotional responses of patients and their families, moral distress, and cautionary tale of nurses; (3) Financial outcomes: imposing costs on the patient and financial loss of the nurse; (4) Organizational outcomes: the normalization of misconduct, chaos in the organization, waste of the organization’s resources, and reputational damage to the organization.

**Conclusion:**

Professional misconduct by nurses can have adverse outcomes for patients in physical, mental, and financial dimensions, their families, nurses, and healthcare organizations. Therefore, it is indispensable to adopt management strategies to reduce the rate of professional misconduct.

**Supplementary Information:**

The online version contains supplementary material available at 10.1186/s12912-024-01859-3.

## Background

Nurses play a vital role in ensuring patients’ well-being and recovery. They are patients’ trustworthy caregivers, advocates, and instructors [1]. According to the code of ethics for nurses, they have the responsibility for protecting the patient, society, and the profession against possible harm [2]. Maintaining nurses’ ethical standards and professional conduct is imperative in ensuring patient safety, trust, and integrity of the nursing profession [1, 3, 4]. In the nursing profession as a caring and humane profession, there is the possibility of another reality termed professional misconduct [5–7]. Professional misconduct refers to any practice or action by nurses that deviates from the established ethical and professional standards and guidelines [8, 9].

Professional misconduct in nursing is a multifaceted issue with wide-ranging outcomes in patient safety (physical and mental harm or even death), trust in the healthcare system, and healthcare workers’ well-being. Professional misconduct can damage the profession’s reputation and weaken nurses’ vital role in society [10, 11].

Considering that professional misconduct weakens the integrity of nursing practice, perceiving consequences is of particular importance for nurses, healthcare managers, and policymakers since it emphasizes the prominence of maintaining the highest professional standards [12]. As part of a broader research initiative, this study extensively examines the multifaceted repercussions of such misconduct, extending beyond immediate effects, to deepen understanding across various dimensions. Given healthcare organizations’ mandate to deliver high-standard care with minimal harm, comprehending these implications becomes paramount. The research enhances related knowledge by categorizing the consequences of misconduct, highlighting potential dangers and irreparable harm. Also, it emphasizes the imperative of responsibility and ethical conduct to enhance patient quality and safety. Through this endeavor, the study addresses gaps in understanding within the field.

## Methods

### Study design, setting, and participants

This qualitative descriptive study was conducted through the generic qualitative design and content analysis approach to data analysis. The study setting was different wards of general and referral hospitals in the capital of one of the western provinces of Iran. The participants included in the study were selected using purposive sampling. The inclusion criteria included at least a bachelor’s degree in nursing, an experience of observing professional misconduct by colleagues, and the willingness to discuss experiences. Before conducting the interview, the researcher coordinated the interview time and location while establishing communication with the eligible participants and explaining the study objectives. In order to achieve maximum diversity and richness of data, participants with diverse personal and professional characteristics were selected. Sampling continued until saturation, resulting in the inclusion of 22 nurses and nursing managers aged between 25 and 48 years (Table 1).

**Table 1:** Demographic data of the participants

participants	gender	age (in years)	education level	work experience (in years)	position
1	Male	38	Master’s	13	Clinical Nurse
2	Female	39	Bachelor’s	16	Clinical Nurse
3	Female	47	Undergraduate	24	Clinical Nurse
4	Female	48	Bachelor’s	24	Clinical Nurse
5	Female	47	Bachelor’s	25	Clinical Nurse
6	Female	47	Bachelor’s	22	Clinical Nurse
7	Female	39	Bachelor’s	15	Clinical Nurse
8	Female	26	Bachelor’s	3	Clinical Nurse
9	Female	37	Bachelor’s	14	Clinical Nurse
10	Male	42	Bachelor’s	18	Head Nurse
11	Female	49	Bachelor’s	21	Clinical Supervisor
12	Male	27	Bachelor’s	2	Clinical Nurse
13	Male	41	Master’s	19	Nursing service manager
14	Male	37	Bachelor’s	15	Clinical Nurse
15	Female	36	Bachelor’s	14	Clinical Nurse
16	Female	37	Master’s	14	Clinical Nurse
17	Female	29	Bachelor’s	1	Clinical Nurse
18	Male	26	Bachelor’s	3	Clinical Nurse
19	Female	25	Bachelor’s	2	Clinical Nurse
20	Female	31	Master’s	4	Clinical Nurse
21	Female	45	Bachelor’s	22	Clinical Nurse
22	Female	35	Bachelor’s	12	Clinical Nurse

### Data collection

After preparing the interview guide using the expert opinions of the research team, the data were collected through a semi-structured individual interview. Each interview lasted between 45 and 90 min. Data collection was performed by the first author under the supervision and cooperation of the research team. Participants were asked: “Describe your experience on the outcomes of professional misconduct.” “Who is affected by the outcomes of professional misconduct? Explain it.” At the end of the interview, open questions were asked. The interviews were recorded using a mobile phone with the participants’ permission. Data collection and analysis were performed simultaneously from February 2021 to August 2021.

### Data analysis

The conventional content analysis method was employed by following five steps proposed by Graneheim and Lundman [13]: (1) Implementing the entire interview immediately after each interview, (2) Reading the entire text several times to get an overall understanding of its content, (3) Determining semantic units and basic codes, (4) Classifying primary codes in more comprehensive categories, and (5) Determining the main theme of categories.

Interviews were recorded and transcribed using Word software, followed by iterative readings for content understanding. Semantic units were identified based on study objectives, and primary codes were derived. The initial codes were categorized, and the main and sub-themes were determined. Data management was facilitated by MAXQDA10 software.

It is noteworthy that the researcher, aimed to maintain objectivity during the coding process by closely aligning the codes with the data, and setting aside personal biases and preconceptions.

### Trustworthiness

The following strategies were used to establish the trustworthiness of Study [14] Credibility was achieved through trust-based communication and prolonged engagement with the participants and the data and by providing a lot of time for data collection. Dependability was ensured by checking the consistency between quotes and codes/subthemes by the research team and two external observers familiar with qualitative research. In addition, confirmability was established by presenting the quotes extracted from each interview and returning the text of several interviews to a number of participants and applying their opinions, Transferability was enhanced by selection of participants with maximum diversity in terms of age, gender, work experience, educational level and position and detailed description of the research process, participant characteristics, and study context. In addition, quotes were expressed directly by providing each participant’s quote (P).

### Ethical considerations

The Joint Ethics Committee of the Faculty of Nursing, Midwifery, and Rehabilitation of Tehran University of Medical Sciences approved this study with the ethics code IR.TUMS.FNM.REC.1400.187. The study objectives were explained to the participants at the beginning of the interviews. Due to the disapproving nature of professional misconduct and the sensitivity of the issue, the possibility of voluntary participation, confidentiality, and anonymity of individuals and their organizations were guaranteed. Written informed consent was obtained from all participants. Transcripts were securely stored in an encrypted file on a personal computer and destroyed following data analysis to further protect the confidentiality of participants.

## Results

The outcomes of professional misconduct by nurses were categorized into four main themes and 11 sub-themes (Table 2).

**Table 2:** The main themes and sub-themes extracted from the data

Main Themes	Sub-themes
Physical outcomes	Critical threat to patients
	Weakening patients’ safety
Psychological outcomes	Psycho-emotional responses of patients and their families
	Moral distress of nurses
	Cautionary tale
Financial outcomes	Imposing costs on the patient
	The financial loss of the nurse
Organizational outcomes	The normalization of misconduct
	Chaos in the organization
	Waste of the organization’s resources
	Reputational damage to the organization

### Physical outcomes

Data analysis indicated that patients were the primary individuals affected by professional misconduct by nurses and experienced more harm than other parties. One of the most critical outcomes of misconduct is the physical impact on patients. This theme is subdivided into two aspects: critical threat and undermining of patient safety.

#### Critical threat to patients

The participants’ experience showed that professional misconduct by nurses exposes patients to critical and adverse events such as death, disability such as leg amputation, and critical injuries such as pneumothorax, finger gangrene, tissue necrosis, burns, bleeding, and falls.

“*The patient was critically ill and we announced the CPR code ten minutes after the shift was handed over. We checked and noticed that they hadn’t inserted an IV line for him. It was impossible to do it with a blood pressure of 65. We finally inserted the intravenous) IV (, but it was in vain…”* (Participant 9).

#### Weakening patients’ safety

According to the participants’ experiences, in addition to critical injuries, less life-threatening injuries such as mouth sores and infections could occur following professional misconduct by nurses. There was also the possibility of unwanted side effects. Yet most of these complications may not appear right away and be noticed after discharge from the hospital.

“*One of the colleagues, as she said, made a potion, combined several antibiotics into the Microset, and injected it into the patient*.” (Participant 14).

This theme shows that considering the physical aspect, professional misconduct by nurses ultimately leads to a decrease in the quality of care and safety and delays the treatment process.

### Psychological outcomes

Based on data analysis, professional misconduct by nurses affects psychological aspects in addition to physical dimensions. This outcome may involve not only patients but also their families and nurses. This theme includes the psycho-emotional responses of patients and their families, moral distress, and edification of nurses.

#### Psycho-emotional responses of patients and their families

Nurses’ experiences showed that professional misconduct sometimes caused psycho-emotional reactions in the patient or their companions. These side effects were reported as crying, feeling abandoned, distrust, dissatisfaction, cursing, aggression, objection, reporting to the authorities, and complaints.

“*At the beginning of the outbreak, a patient suspected of being infected with coronavirus was hospitalized in the ward and was left in the room; the door was closed. She was ordered not to get out of the room because she could spread the coronavirus to other patients; she was crying all the time.”* (Participant 11).

#### Moral distress of nurses

Sometimes, the repercussions of professional misconduct by a nurse affect both the perpetrator and the cooperating and witnessing nurse. The participants stated that, at times, they experienced various emotional reactions, including discomfort, remorse, guilt, and even psychological complications and quitting work after committing misconduct. In addition, the colleagues of a nurse who is the perpetrator of the misconduct may express regret, discomfort, and anger upon witnessing this situation.

“*I know that catheterization is a sterile procedure, and I’m fully aware of it, but at that moment, there may not be a betadine or a sterile set or gloves. I may not do it correctly and scientifically as I should, which is really sad. Most of the time, we feel guilty.”* (Participant 10).

#### Cautionary tale

According to some participants’ experiences, the effect of a nurse’s encounter with a colleague’s misconduct depended on the morale and personality of the witnessing nurse. By witnessing misconduct and its negative outcomes for the patient and the nurse committing it, the nurse may learn never to commit such misconduct. According to the famous quote, “A man profits more by the sight of an idiot than by the orations of the learned,” it should also be instructive and improve patient care quality.

“*Misconduct by a colleague can influence the nurse seeing it and make them improve, that is, not perform that wrong deed. Due to a written warning to a few colleagues because of the rapid infusion of antibiotics, the others learned and are now very careful*.” (Participant 10).

This theme showed that professional misconduct by nurses might harm patients not only physically but also mentally and occasionally cause psychological problems for the nurses. It should be noted that, besides all the negative outcomes, professional misconduct by nurses has a positive consequence, which is a cautionary tale of other nurses and, subsequently, efforts to improve care.

### Financial outcomes

Data analysis showed that another outcome of professional misconduct by nurses was the financial outcomes that could affect the patient or nurse. This theme includes two subthemes: ‘imposing costs on the patient’ and ‘financial loss of the nurse.’

#### Imposing costs on the patient

According to the obtained data, additional costs are imposed on the patient due to adverse events and unwanted complications caused by the reduced care quality resulting from professional misconduct by nurses. These costs may be related to increased length of hospital stay and the need for additional procedures or medication.

“*Unfortunately, some colleagues don’t observe the principles of sterile technique when dressing, which can cause the patient to return with an infection at the surgery site and need to take intravenous antibiotics such as Ciprofloxacin and Clindamycin and be hospitalized for a few days, all of which impose an additional cost to the patient.”* (Participant 11).

#### The financial loss of the nurse

Several participants’ experiences showed that the nurse might experience legal issues such as warnings, reprimands, and referrals to the administrative violations department following committing misconduct, which, especially if repeated, could negatively affect the process of recruiting the training nurses or changing their employment status, in-service promotion process when being appointed to a position until retirement. Based on the participants’ experiences, addressing misconduct might have financial outcomes for the nurse who committed it. These financial damages included a deduction of salary and wages, no further promotion after warning, reprimand and its negative impact on salary, and incurring damages.

*“Our nurse colleague hadn’t paid attention to the warmer’s temperature. The mask on the baby’s nose was almost burnt and caused nasal necrosis. The baby’s family pursued it. The nurse was fined to pay the damages. ”* (Participant 8).

This theme generally indicated the financial damages resulting from professional misconduct by nurses, which might affect patients and nurses.

### Organizational outcomes

Professional misconduct by nurses has negative outcomes not only for individuals but also for the organization. This theme includes the subthemes of normalization of misconduct, chaos in the organization, waste of the organization’s resources, and reputational damage to the organization.

#### The normalization of misconduct

Participants stated that one of the organizational outcomes of misconduct was its normalization for the perpetrator, modeling, and contagion of misconduct to other colleagues, leading to the normalization of erroneous conduct in the organization.

“M*isconduct possibly affects others as well, as it is considered a routine, as they think somebody did it, and there was no problem. Now, in ward X, it has become routine that vital signs aren’t monitored and are only recorded*.’ (Participant 10).

#### Chaos in the organization

The data analysis showed that due to professional misconduct by nurses, colleagues might be forced to compensate for their colleague’s misconduct by carrying out the medical orders for the maltreated patient. As a result, nurses usually avoided working shifts together with that nurse. There might also be turmoil, chaos, arguments, protests, complaints, and even physical encounters between patients and their companions with the medical staff or colleagues.

“*My colleague’s work burden falls on my shoulders, so I should also manage her duty. For example, in my shift, I followed up on a medicine that had to be prepared in the previous shift and made a prescription for the patient; the patient prepared it but growled at me because the medicine was expensive. The doctor talked to me as if I was the one who hadn’t done it while it hadn’t been followed up in the previous shift*.” (Participant 19).

“*Colleagues who impatiently do the patient’s tasks get angry at the patient. The patient or the companion asks one question or two; upon the third question, they conflict with the patient’s companion. We have a code called code 44 for a security guard, which is often announced during their shifts.”* (Participant 19).

#### Waste of the organization’s resources

After analyzing the data, it was revealed that due to the professional misconduct by nurses, the patient might need a transfer to the intensive care unit or more specialized centers, additional procedures, such as debridement, intubation, dialysis, surgery, re-surgery, or cancellation of surgery, increased hospital stay, and re-hospitalization. By jeopardizing the quality and safety of patient care, these cases lead to complications, and managing them can impose additional costs on the hospital. Some participants believed that failure to provide optimal care caused the patient’s condition to aggravate and the nurse’s workload to increase.

“*The patient, who was just discharged from the operating room, was bleeding badly. The nurse hadn’t followed up or informed the doctor. The patient was transferred to the ICU due to severe bleeding and was treated for approximately 15–16 days. He was operated on twice*.” (Participant 14).

In addition, nurses’ professional misconduct directly leads to the waste of resources and equipment.

“*For example, in the COVID-19 situation, when the equipment and supplies were scarce from the beginning, they rationed it for the wards. A male colleague poured Septicidine. Well, it was wasted. It could be used in the COVID-19 ward*.” (Participant 7).

#### Reputational damage to the organization

According to the data analysis, professional misconduct could lead to damage to the reputation and credibility of the nursing profession and loss of public trust in nurses and healthcare organizations in general.

“*Sometimes we refer the patient to a certain hospital, but they say they wouldn’t go there even if they die. They believe whoever is referred to that hospital won’t stay alive*.” (Participant 13).

This theme revealed outcomes of misconduct that threatened and affected the healthcare organization.

## Discussion

In the present study, nurses’ experiences regarding the outcomes of professional misconduct were investigated. The results showed that this phenomenon had widespread outcomes in different dimensions and levels, including patients, nurses, and healthcare organizations. In line with the present study, researchers concluded in a systematic review that unprofessional conduct included multidimensional issues and serious outcomes concerning patient safety, nurses, colleagues, managers, and healthcare organizations [6].

One of the significant outcomes of professional misconduct is physical outcomes, which can critically threaten patients’ health and life or jeopardize their safety. In a review, the threat to patients’ safety has been identified as the main reason for adopting disciplinary measures against nurses [15]. In addition, in a qualitative study, various unsafe practices leading to physical harm to patients have been identified and classified [16]. In line with the present study, Rooddehghan et al. (2018) reported that missed nursing care could lead to the elimination or postponement of scheduled therapies, which causes serious life threats, complications, and, as a result, patient dissatisfaction [17]. Professional misconduct in health care can jeopardize patients’ safety, health, and well-being [5, 18, 19]. Since the main goal of health care is to provide quality and safe care to patients, the physical outcomes of professional misconduct by nurses are considered the most important outcomes, and their prevention is absolutely vital.

Another consequence of professional misconduct by nurses is its psychological effects on patients and nurses. Healthcare workers’ misconduct can cause psychological harm to patients, including anxiety, feeling insulted, and fear [18]. Moreover, misconduct demonstrates the violation of patients’ human rights and dignity [7]. Since the quality of services provided to patients is an important component of their satisfaction [20], professional misconduct can reduce patient satisfaction by negatively affecting the quality of care. Nurses charged with professional misconduct face a variety of outcomes, including psychological, physical, and mental suffering [21]. Furthermore, observing misconduct can lead to moral and emotional distress, sympathy for patients, and increased negative emotions such as distress, sorrow, guilt, bias, and negative stigma in fellow nurses [18, 22]. These nurses may to leave their positions and may experience anxiety, sleep disturbances, and uncertainty in dealing with their colleagues [16]. In general, professional misconduct can cause psychological problems not only for patients but also for nurses, which supports the need for prevention and corrective action.

Unprofessional conduct is a complex phenomenon that impacts nurses’ practice [6]. In the present study, it was found that professional misconduct could serve as edification for other nurses. In other words, misconduct by colleagues can serve as a cautionary tale to assist nurses in improving their performance. In this regard, studies have shown that unsafe practices by colleagues and related complaints can provide an opportunity for nurses to strengthen their abilities by focusing more on themselves and being more attentive, and contribute to professional development and increased patient safety [16, 23]. Therefore, it seems that, when encountering colleague misconduct, nurses can use negative experiences in the organization and enhance their skills and precision in order to improve professional conduct and patient safety.

Other outcomes of professional misconduct by nurses obtained in the present study were financial outcomes that could affect patients and nurses. In line with this finding, a study shows that unsafe practices can impose additional costs on patients [24]. The financial losses of nurses caused by professional misconduct can be related to legal outcomes such as restrictions, suspension, revocation of professional license, or finement [15, 25, 26]. It can be concluded that, regarding economic issues, professional misconduct by nurses can harm the patients and even the nurses.

Professional misconduct by nurses has outcomes not only for patients and nurses but also for the healthcare organization. These outcomes include issues such as the normalization of misconduct, chaos in the organization, waste of the organization’s resources, and reputational damage to the organization. These issues can reduce the organization’s efficiency and cause concerns about the safety and quality of services provided by nurses. Professional misconduct is often initiated by one individual; however, it can spread quickly, change the organization’s dominant values, norms, and behaviors, and become established [27]. These disciplinary processes affect the nursing profession, and these impacts become more significant in retaining nurses, particularly in global staffing shortage conditions [25]. In addition, professional misconduct in health care can jeopardize the quality of nurses’ teamwork [5], increase colleagues’ workload [22], and threaten the organization’s long-term credibility and ultimate sustainability by deviating the organization from achieving its main goals [10]. Misconduct in health care can cause patients and the general public to mistrust medical affairs and damage the reputation of the nursing profession and the organization [24, 28, 29]. Jeopardizing satisfactory standards of practice is a clear violation of nursing ethics, norms, and laws, particularly public trust in nurses and the nursing profession as a whole [30]. To prevent these challenges, the organization can create a safety culture, develop protocols to report misconduct, and encourage and support nurses. These measures can prevent misconduct, help increase public trust in the nursing profession, and improve the working conditions of nurses.

The present study had several limitations. At first, some participants had doubts about the confidentiality of their names and institutional information in the study. This concern was resolved by assuring them about the anonymity and confidentiality of the information. This research was conducted qualitatively, and therefore, the generalizability of the findings is limited.

## Conclusion

According to the results of this study, it is revealed that the outcomes of professional misconduct in the nursing field affect not only patients and nurses but also the healthcare organization. The outcomes of professional misconduct have diverse and widespread dimensions. Physically, professional misconduct can lead to a critical threat to patients or jeopardize their safety. Psychologically, it can create psychological responses in patients and nurses or become an edification for other nurses. Financially, it might impose costs on patients and financial losses for nurses. Organizational effects include the normalization of misconduct, chaos in the organization, waste of resources, and damage to the dignity and credibility of the organization. To prevent these complications, there is a need for programs and management measures to deal with professional misconduct and ensure the provision of safe, quality, and compassionate care to patients. To reduce serious outcomes, further studies in diverse nursing communities are required.

### Electronic supplementary material

Below is the link to the electronic supplementary material.

Supplementary Material 1

## Data Availability

Availability of data and materials: Data are available by contacting the corresponding author.

## Abbreviations

CPR: Cardiopulmonary resuscitation
IV: Intravenous
ICU: Intensive care unit
COVID-19: Coronavirus disease 2019
