# Supplementary material for: Outcomes of professional misconduct by nurses: a qualitative study
Source: BMC Nurs. 2024 Mar 25;23:200. doi: 10.1186/s12912-024-01859-3 (PMC10962125; doi:10.1186/s12912-024-01859-3)
Supplement: Supplementary file 1 — Supplementary Material 1 [file 12912_2024_1859_MOESM1_ESM.docx]

**Interview Guide**

Describe your experience on the outcomes of professional misconduct.

Who is affected by the outcomes of professional misconduct? Explain it.

**Then exploratory questions such as:**

Can you explain more about this?

What do you mean by that?

Is there anything else you would like to explain?
